# Towards Strong Inference in Research on Embodiment – Possibilities and Limitations of Causal Paradigms

**DOI:** 10.5334/joc.139

**Published:** 2021-01-08

**Authors:** Markus Ostarek, Roberto Bottini

**Affiliations:** 1 Max Planck Institute for Psycholinguistics, Wundtlaan 1, 6525XD Nijmegen, The Netherlands; 2 Center for Mind/Brain Sciences (CIMeC), University of Trento, Italy

**Keywords:** Embodied cognition, Semantics, Neuropsychology, Action and perception

## Abstract

A central question in the cognitive sciences is which role embodiment plays for high-level cognitive functions, such as conceptual processing. Here, we propose that one reason why progress regarding this question has been slow is a lacking focus on what Platt (1964) called “strong inference”. Strong inference is possible when results from an experimental paradigm are not merely consistent with a hypothesis, but they provide decisive evidence for one particular hypothesis compared to competing hypotheses. We discuss how causal paradigms, which test the functional relevance of sensory-motor processes for high-level cognitive functions, can move the field forward. In particular, we explore how congenital sensory-motor disorders, acquired sensory-motor deficits, and interference paradigms with healthy participants can be utilized as an opportunity to better understand the role of sensory experience in conceptual processing. Whereas all three approaches can bring about valuable insights, we highlight that the study of congenitally and acquired sensorimotor disorders is particularly effective in the case of conceptual domains with strong unimodal basis (e.g., colors), whereas interference paradigms with healthy participants have a broader application, avoid many of the practical and interpretational limitations of patient studies, and allow a systematic and step-wise progressive inference approach to causal mechanisms.

## 1. INTRODUCTION

According to many modern theoretical accounts, cognition and bodily experience are strongly intertwined. Embodied views of cognition propose that the sensory-motor systems are recycled for high-level cognitive functions (Barsalou, 1999, 2008; Gallese & Lakoff, 2005; Meteyard et al., 2012; Pulvermüller, 2005). Here, we discuss how causal paradigms can evaluate the key claim that sensory-motor simulations contribute functionally to high-level cognitive processing. Before we begin, two things are in order. First, we need to specify what we mean by “sensory-motor simulation”. We view simulation as the activation of sensory-motor processes during high-level cognitive processing. Simulations are a constructive process, meaning that rather than being re-activations of past sensory states they reflect the context-dependent re-use of sensory-motor processes (e.g., Barsalou, 1999; Kiefer & Pulvermüller, 2012). As such, experiential sensory traces are the ingredients from which simulations can be formed. This makes simulations generative; you can think about a dragon even if you have never seen one. This view of simulation reflects what we take to be the central proposal of many of embodied accounts that have been developed (Barsalou, 1999; Kiefer & Pulvermüller, 2012; Meteyard et al., 2012). Second, we need to specify what it means for simulations to “contribute functionally” to a task. Simulations are considered functionally relevant for a task if it can be demonstrated that the failure to properly simulate leads to a measurable negative effect on task performance. We use the term “causal paradigm” to refer to paradigms which test functional relevance. Here, we discuss the potential and limitations of three types of causal paradigms: experiments capitalizing on congenital sensory-motor disorders, experiments capitalizing on acquired sensory-motor disorders, and interference paradigms with healthy participants.

### 1.1. TOWARDS STRONG INFERENCE

The motivation for this paper was to think about ways in which progress in our understanding of the role of sensory-motor processes in high-level cognition can be accelerated. In different shapes and forms, it has been recognized by several authors that the field is presently stuck in a sort of impasse (Mahon, 2015; Ostarek & Huettig, 2019; Zwaan, 2014). It is likely that multiple factors have caused this situation. Here, we concentrate on one factor that we consider particularly important, namely the lacking focus on what Platt called “strong inference” (Platt, 1964). Strong inference relies on the systematic application of a set of steps geared towards minimizing the negative effects of researchers’ biases and thereby maximizing scientific progress. It includes three main steps: 1) Devising alternative hypotheses. Rather than simply focusing on support for hypotheses that one sets out to test, Platt argued that it is crucial to focus on hypotheses that can be *excluded*. 2) Devising a crucial test. Tests are considered crucial if they exclude on or more of the alternatives that were generated in the first step. 3) Carrying out the experiment(s) properly. These three steps are then repeated in order to refine the remaining hypotheses and converge on the most likely conclusion.

In contrast to this ideal scenario, many results that are commonly cited as evidence for embodied theories of cognition are equally consistent with amodal accounts (Mahon & Caramazza, 2008; Ostarek, Joosen, et al., 2019; Ostarek & Huettig, 2019; Pylyshyn, 1973), making it impossible to adjudicate between the two models. This *consistency fallacy* (i.e. the lacking focus on the exclusion of alternative explanations) is apparent, for instance, in congruency effects in many behavioral paradigms (e.g., Glenberg & Kaschak, 2002; Stanfield & Zwaan, 2001; Zwaan et al., 2002), which provide at best indirect evidence for the reenactment of motor and perceptual programs, and can equally be accounted for (although they might not have been predicted; Barsalou, 1999) by symbolic models of cognition (Fodor, 1975). For instance, in a commonly used paradigm (Zwaan et al., 2002), participants process sentences that imply objects to have a particular shape (e.g., *the ranger saw an eagle in the sky*). Just after the sentence, they see a picture and have to decide whether it is an object that was mentioned in the sentence or not. Crucially, in trials where an object is presented that was mentioned in the sentence (an eagle), it either matches (outstretched wings) or mismatches (closed wings) the shape implied in the sentence. Researchers typically observe shorter reaction times (RTs) in the shape matching condition, which has been cited as evidence that sentence processing involves perceptual simulations of objects shapes (Zwaan et al., 2002; Zwaan & Pecher, 2012). In particular, the idea is that sentence comprehension activates visual simulations that then facilitate visual processing of matching pictures. However, this interpretation relies on a leap of faith because the result of shorter RTs is equally compatible with amodal views (for discussion see Ostarek & Huettig, 2019; Ostarek et al., 2019). On such accounts, the shape match effect can simply be explained as a priming effect in an amodal conceptual system; shape information extracted from the sentences and from the target pictures is processed in an amodal system and informational congruency leads to more efficient processing. Congruency paradigms of this sort can tell us something about the informational contect that is activated during language comprehension and conceptual processing, but they are not decisive when it comes to the question of embodiment. The reason why is that they do not exclude any of the contentious alternatives.

It is important to note that congruency paradigms do not inherently have these limitations. Measures can be taken to avoid situations of interpretational ambiguity. One promising solution is to create situations in which stimuli that language is predicted to interact with are only processed at the level of interest (Ostarek & Huettig, 2019). For instance, continuous flash suppression can be used in a way that probes the influence of words on basic visual processing in the absence of conceptual processing of the target picture (Ostarek & Huettig, 2017b).

**Figure 1:**
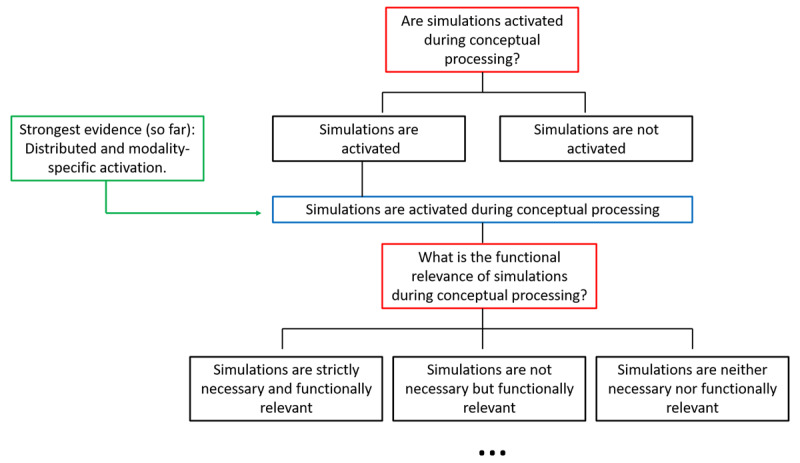
The first part of a logical tree illustrating how multiple hypotheses can be sequentially explored in order to zero in on the most important questions.

A useful tool to avoid situations where evidence fails to exclude alternative explanations suggested by Platt is that of a logical tree. Logical trees can force us to systematically think about alternative hypotheses and come up with crucial tests. For instance, the tree presented in ***Figure 1*** starts out with the broad question of whether simulations are at all activated during conceptual processing. This question has been addressed extensively with neuroimaging methods and there generally appears to be consensus that they often are (e.g., Kiefer & Pulvermüller, 2012; Meteyard et al., 2012). For instance, the evidence suggests that words referring to actions and visual objects activate the corresponding action and perceptual systems (e.g., Fernandino et al., 2016; Hauk et al., 2004; Lewis & Poeppel, 2014; Shtyrov et al., 2014; Tettamanti et al., 2005).

### 1.2. ZEROING IN ON THE FUNCTIONAL ROLE OF SIMULATIONS

Once we confirmed the hypothesis that simulations are activated during conceptual processing (and excluded the alternative), we can go on to ask whether simulations have functional roles in conceptual processing and understand what these are. It is at this level that many theoretical accounts differ. For instance, the “distributed neuronal assemblies theory” (Pulvermüller, 1999; 2005) proposes that links between word forms and sensory-motor states are formed via Hebbian learning. Thus, whenever a word (such as *kick*) is processed, associated (leg movement related) motor processes automatically activate and contribute to word comprehension, as they have become a part of the neuronal assembly involved in processing the word. Other theories, such the “perceptual symbols systems theory” (Barsalou, 1999), instead envision a more flexible retrieval mechanism. On this view, perceptual symbols are formed by selectively attending to aspects of our sensory-motor experience and are organized in simulators that integrate related perceptual symbols. Simulators construct simulations that are strongly shaped by contextual factors that constrain which aspects of conceptual knowledge are activated in a given moment. According to the “grounding-by-interaction model” (Mahon & Caramazza, 2008), the functional role of the sensory-motor systems for conceptual processing is limited. Here, the idea is that concepts are represented in amodal symbols, which are connected to sensory-motor systems and interact with them. Due to the flow of information between these different systems, they can influence each other. However, conceptual knowledge is not predicted to depend on the sensory systems, even though concepts may be “isolated” or “impoverished” when grounding is not possible (Mahon & Caramazza, 2008). On the other extreme, fully amodal theories (Fodor, 1975; Pylyshyn) propagate a view of the conceptual system that is completely symbolic and propositional: Once sensorimotor information is transduced in symbols, only the latter constitute the “stuff” of thought.

Without going into the details of each theoretical proposal (which is well beyond the scope of this review), the problem of the functional relevance of simulations can be approached by means of a logical tree with progressively more specific alternative hypotheses. Once it is established that sensorimotor processes are activated during high level cognition, three alternative hypotheses are at stake: simulations may be strictly necessary and functionally relevant; they may not be strictly necessary but have functional relevance when they are in place; or they might be neither necessary nor functionally relevant. By “strictly” necessary, we mean that without the ability to simulate conceptual processing is entirely impossible. On that view, simulations would be necessary at any level of conceptual processing: e.g., from the effortless activation of a word’s meaning, to the delibrate retrieval of conceptual content from memory.

Causal experiments based on the consequences of congenital sensory-motor disorders, or experiments capitalizing on acquired sensory-motor disorders, as well as interference paradigms with healthy participants are priviledged research tools to answer this type of question. At first sight, predictions may seem straightforward: if simulations are strictly necessary to understand action words, then people with congenital motor impairments should have difficulties with them, etc. However, as will become clear from the next section, things are not that simple. It will turn out to be surprisingly difficult to convincingly exclude any of the three alternative hypotheses of our logical tree based on the results of experiments on individuals with congenital sensory-motor disorders. This will however be crucial for the field to advance. The analysis below is meant to draw attention to the theoretical potential and the limitations of causal paradigms that have been used and more importantly to encite researchers to follow the principles of strong inference in future investigations.

## 2. CAUSAL PARADIGMS

### 2.1. CONGENITAL SENSORY-MOTOR DEFICITS

#### 2.1.1. Congenital motor disorders

Embodied theories of cognition assume that experience-dependent sensory-motor simulation is crucial for high-level cognition. An obvious test of this assumption is the study of individuals with atypical sensory-motor experience, such as people born without upper limbs. The rationale of many studies was that if sensory-motor experience is crucial for high-level cognition, then these individuals should present with measurable deficits in tasks that are predicted to rely on sensory-motor simulation. Conversely, if they perform at the same level as healthy control participants one can conclude that sensory-motor simulation is not necessary for the task.

One relevant line of research used the hand laterality judgement task (HLJ), a variant of the mental rotation paradigm (Cooper & Shepard, 1975). In the HLJ, participants see hands rotated at different angles and are asked to decide whether it is a left or right hand. The two crucial findings in this paradigm were that 1) RTs are strongly influenced by how hard it would be for participants to put their own hand in the displayed position (Parsons, 1987; 1994) and 2) a handedness effect is usually observed (right-handers respond faster to rotated pictures of right hands). The effect of biomechanical constraints on task performance was taken as evidence that participants rely on motor simulations to solve the task. In line with this view, there is evidence that effects of biomechanical constraints are not observed in participants with right hemiparesis (Craje et al., 2010; Mutsaarts et al., 2007). Furthermore, these patient groups, as well as individuals born with one or two missing hands (Funk & Brugger, 2008) typically respond much more slowly (~1s, using their unaffected hand) than controls (Craje et al., 2010; Steenbergen et al., 2007; Van Elk et al., 2010). Finally, responses to pictures of the missing hand are often slower than responses to pictures of the preserved hand (Craje et al., 2010; Funk & Brugger, 2008; Van Elk et al., 2010). The finding that motor deficits lead to altered performance on these tasks suggests that the motor system can be functionally relevant for them.

However, challenging the view that motor simulation is strictly necessary for effects of biomechanical constraints to emerge, such effects have been reported in participants with congenital hemiparesis (brain damage leading to the inability to move the left or right limbs) (Steenbergen et al., 2007) and in individuals born without upper limbs altogether (Funk & Brugger, 2008; Vannuscorps et al., 2012; Vannuscorps & Caramazza, 2015). Moreover, in tasks that are arguably more complex than simply deciding whether one is seeing a left or right hand, the evidence points to even smaller consequences of congenital motor disorders. Individuals born without hands were found to carry out predictive eye movements when observing hand actions (Vannuscorps & Caramazza, 2017), and more generally to comprehend and memorize hand actions just like typically developed participants do (Vannuscorps & Caramazza, 2016b). Moreover, in neuroimaging studies they were found to activate a highly similar network of brain regions during action observation (Striem-Amit et al., 2017; Vannuscorps et al., 2019; Vannuscorps & Caramazza, 2016b). One study, however, observed impaired short-term memory for actions in this group compared to controls (Vannuscorps & Caramazza, 2016a), suggesting that without motor simulations it is harder to maintain information about specific hand postures in working memory.

In sum, the evidence for the functional relevance of motor simulations in high level cognition is mixed: the effects of congenital motor disorders range from making no difference at all (Vannuscorps et al., 2012; Vannuscorps & Caramazza, 2016b) to completely abolishing effects due to biomechanical constraints (Craje et al., 2010; Funk & Brugger, 2008; Steenbergen et al., 2007). However, it is clear that the evidence does not support a scenario where motor simulation is strictly necessary for action cognition. Crucially for the current analysis, this kind of evidence cannot falsify the more general hypothesis that some kind of simulation (not necessarily motoric) is strictly necessary for high level cognition. This is because concepts tend to be multimodal in the sense that they are associated with experience in multiple modalities (e.g. Fernandino et al., 2015). For instance, actions can be associated with one’s own motor and somatosensory experience, but also with the visual experience of seeing others performing them. In support of this hypothesis, a meta analysis of fMRI studies showed that responses in and around visual motion-sensitive, but not premotor and motor regions, are consistently observed during action concept processing (Watson et al., 2013; see also Bottini et al. 2020). This suggests that even for typically developed individuals, visual experience dominates conceptual processing of actions. It is conceivable that in individuals born without arms, spared visual-spatial computations (which may be enhanced for compensation) can go a long way such that behavioral performance is typically affected only slightly (rather than catastrophically). After all, experience dependence forms the core of embodied accounts. The things that one can simulate are constrained by the things one has experienced. When a particular type of experience (e.g., motoric) is not available to a person, that does not mean that others aren’t (e.g., visual). From that perspective it would not be surprising if people born without hands behave similarly to people with hands in tasks for which visual (or other types of) experience can suffice. For instance, there is no a priori reason why mental rotation of hands has to involve motor simulations, and it is conceivable that such a task can be solved via visual-spatial processes (see Barsalou, 2016 for a similar argument). Thus, studies involving congenital motor disorders constitute evidence against the hypothesis that intact *motor simulations* are strictly necessary for high-level action-related cognition (Vannuscorps et al., 2012; Vannuscorps & Caramazza, 2016b), but they cannot rule out the more general hypothesis that some kind of simulation is.

Thus, although we have made undeniable progresses in defining the scope and implication of motoric simulations (Vannuscorps et al., 2012; Vannuscorps & Caramazza, 2016b), we are still stuck with all three alternatives in the second level of our logical tree. Can we take the next step and provide strong inference at this logical level, based on the study of congenital sensorimotor disorders? One possibility consists in studying unimodal concepts. Whereas for multimodal concepts alternative experiential channels are available when one is missing, unimodal concepts’ referents can only be directly experienced in one modality. For instance, color can only be experienced visually. A clear prediction from the perspective of experience-based accounts is that differences in conceptual knowledge and/or processing should be strongest when sensory experience of unimodal concepts is precluded. Studying unimodal conceptual domains is a strong test of the experience-dependence of the conceptual system because compensation via alternative modality-specific information can be ruled out. The next section discusses whether congenital blindness results in measurable processing differences for unimodal vs. multimodal concepts.

#### 2.1.2. Congenital blindness

In De Anima (Hicks, 2015), Aristotle envisioned thought to heavily depend on mental images (phantasma) and claimed that it is impossible to think without them. It is a very common intuition (of sighted people) that internal visual processes are irreplaceable components of our conceptual mental life. The case of congenitally blind individuals is in apparent conflict with this intuition. Anecdotally, blind people talk just like sighted people and do not exhibit any signs of lacking conceptual knowledge. In line with this idea, they have been reported to acquire conceptual knowledge that strongly resembles that of sighted people, including knowledge of objects and events that are typically learned via visual experience (Bedny et al., 2019; Bedny & Saxe, 2012; Landau, 1983). According to recent evidence, blind people might even have *enhanced* comprehension abilities when it comes to syntactically challenging sentences (Loiotile et al., 2019). This can be attributed to the fact that the occipital lobe of congenitally blind people is responsive to language and other high-level cognitive functions, such as cognitive control, and thus provides additional processing resources for such functions (Bedny et al., 2011; Loiotile et al., 2019; Van Ackeren et al., 2018). Alternatively, enhanced comprehension of unusually complex sentences could be due to more heavy reliance of language as the unique cue to the meaning of utterances, in contrast to sighted people who often rely on additional (visual) cues (Loiotile et al., 2019).

It is important to note however that a thorough investigation of potential differences between blind and sighted people’s conceptual systems and conceptual processing has not yet been carried out. Some recent efforts have started to fill this gap. In one study (Bedny et al., 2019), blind and sighted participants were asked to rate the semantic similarity of word pairs from different categories which crucially included verbs of light emission (*to sparkle, to glimmer*) and visual events (*to stare, to peak*). The main result was that sighted and blind participants were extremely similar in their ratings, suggesting similar knowledge of important aspects of these verbs’ meanings. On the basis of this result one may claim that we should abandon the branch in the logical tree suggesting that sensorimotor simulations are strictly necessary for conceptual processing. However, an alternative possibility is that visual verbs such as “to glimmer” or “to stare”, can be easily “remapped” onto nonvisual sensorimotor experience because of the correlation between sensory properties. For instance, things that “glimmer” are usually smooth and sharp; and “staring” correlates with muscolar effort and attentional focus, which are all kinds of sensorimotor experiences available to the blind. Thus, blind people may show standard understanding of visual terms because they can remap them onto correlated nonvisual experience and run non-visual simulations. Indeed, as shown by Bedny and colleagues, semantic similarities across these visual verbs were mostly based on the dimensions of intensity (twinkle-blaze) and stability (glow-flicker), which are accessible to blind in other modalities (e.g., audition) and can be based on sensorimotor simulations in the spared senses.

**Figure 2:**
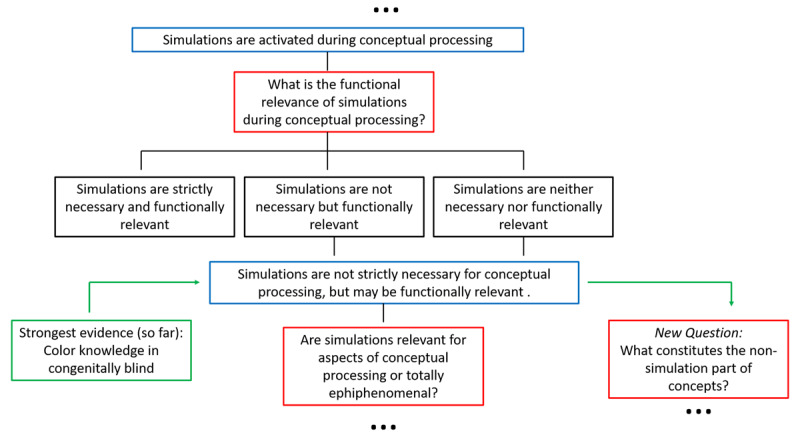
Logical tree illustrating the exclusion of the hypothesis that simulations are strictly necessary for conceptual processing.

A better test-bed for the current purposes would be to investigate conceptual domains or conceptual properties that are not easily re-mappable to non-visual sensorimotor experience and whose meaning is defined by strictly visual properties. A typical example is color. In a study investigating knowledge of animal appearance (size, height, skin texture, color), congenitally blind and sighted controls performed differently (Kim et al., 2019): Congenitally blind people did not appear to know which color animals typically have. Blind people have to learn about typical animal colors from sighted people’s language about animals, which turns out to be rather unreliable, making it surprisingly difficult to extract knowledge of canonical color from sighted people’s utterances (Ostarek, Van Paridon, et al., 2019). Does this result suggest that lack of simulation leads to incomplete conceptual knowledge and that blind individuals have no (or empty) color concepts? In other words, should we conclude that simulations are strictly necessary for conceptual processing? No, we shouldn’t. In fact, there is evidence suggesting that some congenitally blind individuals develop color knowledge that is very similar to that of sighted people. For instance, they know which colors are similar to each other: multi-dimensional scaling of their similarity ratings yields the typical color wheel observed for sighted participants (Saysani et al., 2018). There is recent evidence that blind participants, despite not consistently knowing the typical colors of things, have knowledge about how colors work. For instance, blind participants (just like sighted participants) knew that instances of the same type of fruit (e.g. lemons) tend to have the same color, whereas objects such as cars can have any color (Kim et al., 2020). Moreover, for certain categories and items, such as fruits and vegetables, relatively robust knowledge about typical object color has been reported (Connolly et al., 2007). Indeed it is possible that lower performance in color knwoledge tasks for congenitally blind people is (partly) due to lower exposure to or interest in these concepts. This tendency might be exacerbated for the domain of animals because knowing the typical color of animals is usually inconsequential, as opposed to the domain of fruits where color knowledge is often behaviorally relevant (a green banana is not ripe, a black banana might be rotten). The fact that blind individuals can reach sighted-like levels of performance in color knowledge tasks shows that these limits can be overcome. This suggests that simulations are not strictly necessary for conceptual processing (see ***Figure 2***).

Finally, one last alternative hypothesis should be ruled out. Blind people could rely on idiosyncratic remapping of color knowledge onto sensorimotor experience. For instance, a blind man famously reported by John Locke (1690) described the color “scarlet” as being “like the sound of a trumpet”. In principle, blind people could represent abstract color knowledge by grounding it in concrete sensorimotor experiences (a mechanism suggested also to support abstract knowledge in general; see Borghi et al., 2017). Although the mapping would be idiosyncratic and arguably based on particular subjective experience (as for synesthesic associations), it may still be important and maybe necessary for the development of conceptual representations. At this point, neuroimaging experiments can be helpful to shed some light on this conundrum, because they can assess to what extent conceptual representations involve sensorimotor cortices in sighted and blind. Two studies have approached this issue directly, with convergent results. In one experiment (Bottini et al. 2020) sighted and blind people were asked to judge the similarity of color words (e.g., red, yellow, blue) while undergoing fMRI. Adaptation analysis showed that color similarity was encoded in the visual cortex in the sighted (in a region corresponding to the posterior patch of the V4 complex), whereas it was encoded in superior-temporal (language-related) regions in the blind. Another experiment (Wang et al. 2020) investigated brain regions involved in attributing typical colors to fruits and vegetables. Using representational similarity analysis, the authors showed that objects with similar colors elicited similar pattern of activity in the anterior superior-temporal gyrus in both sighted and blind, whereas the V4 region encoded color similarity only in the sighted. Altogether these results suggest that, instead of being encoded in sensory-motor regions, color knowledge in congenitally blind is consistently encoded in regions that have been associated with amodal/abstract representations (see also Striem-Amit et al. 2018). Thus, although brain imaging studies cannot directly tackle the issue of functional relevance (because they can provide only correlational evidence) they can be important to complement performance-based studies by clarifying the systems that are activated in a given task in a given population. Moreover, they have the intrinsic value of displaying whether different neural architectures are activated during conceptual processing as a consequence of sensory deprivation (Bottini et al. 2020; Wang et al. 2020).

In sum, these results seem to reasonably exclude the possibility that sensorimotor simulation is strictly necessary. Albeit partly based on fMRI data that are correlational in nature and may be insensitive to some important individual differences (Elliott et al. 2020), this appears to be the strongest inference that we can draw from studies on congenital sensorimotor deprivation, allowing us to go a step further in our logical tree. However, to corroborate this inference (that sensorimotor simulations are not strictly necessary for conceptual processing), and to decide whether simulations are completely epiphenomenal or if they, instead, have functional relevance, it will be useful to move beyond paradigms based on congenital sensory deprivation.

### 2.2. ACQUIRED SENSORY-MOTOR DISORDERS

Acquired sensorimotor disorders, due to brain damage, are an interesting testbed in this context. For one thing, when sensory or perceptual regions of the brain are damaged, the possibility of compensation or remapping is minimized. The brain can hardly reorganize completely since it lacks the plasticity of the newborn brain (Murphy & Corbett, 2009), especially in the acute phase of the disorder. Moreover, lack of exposure to a given class of conceptual features (e.g., colors) can be reasonably excluded or considered in the range of variability typical of the standard (non-sensory deprived) population. In principle, this causal model can allow us to corroborate the inference that sensorimotor simulations are not strictly necessary for conceptual processing and disclose whether they are, or not, functionally relevant.

For instance, a growing literature has investigated aspects of conceptual action knowledge in patients with acquired motor disorders. Parkinson’s disease (PD) is known to disproportionately affect the motor system, especially in early stages. Behaviorally, this is reflected in tremors, rigidity, and postural instability (Jankovic, 2008). Neurally, this is reflected in altered physiology in cortical motor regions and the basal ganglia (Herz et al., 2014). A prediction of experience-based embodied accounts is that damage to the motor system should result in deficits in tasks probing conceptual processing of action-related stimuli. In line with this prediction, many studies have reported that motor impairments in PD come hand in hand with action cognition deficits reduced motor simulation abilities (Bocanegra et al., 2015, 2017; Boulenger et al., 2008; Buccino et al., 2017; Cotelli et al., 2007; Fernandino et al., 2013b, 2013a; Garcia et al., 2018; Nisticò et al., 2019; Péran et al., 2003; Roberts et al., 2017; Speed et al., 2017). Importantly, deficits have been shown to disproportionately affect action-related stimuli (Boulenger et al., 2008; Buccino et al., 2017; Fernandino et al., 2013b), to be specific to the limbs that are most affected (Roberts et al., 2017), and they have been observed in patients without general cognitive impairments (Bocanegra et al., 2015, 2017).

Similar results (selective action-specific conceptual deficits) have been obtained in stroke patient with motor impairments (apraxia) (Buxbaum et al., 2005; Negri et al., 2007; Papeo et al., 2010; Pazzaglia, Pizzamiglio, et al., 2008; Pazzaglia, Smania, et al., 2008), in patients with motor neuron disease (Bak, 2013; Bak et al., 2001; Bak & Chandran, 2012; Bak & Hodges, 2004), in patients with progressive supra-nuclear palsy (Bak et al., 2005; Daniele et al., 2013), and in patients with corticobasal degeneration (Cotelli et al., 2006; Silveri & Ciccarelli, 2007; Spatt et al., 2002).

However, this pattern can at least partly be accounted for by confounded factors. For instance, many studies contrasted verbs and nouns which differ in more ways than just their action-relatedness (Silveri et al., 2018). A recent registered report with tightly controlled linguistic stimuli did not obtain evidence for an action-language deficit in PD (Humphries et al., 2019). Moreover, lesions and neuro-degenerative diseases tend to result in rather widespread damage (Herz et al., 2014) making it hard to pinpoint which exact region is associated with which kind of deficit, even though statistical lesion-symptom mapping can be employed to mitigate this issue (Kemmerer et al., 2012; Riccardi et al., 2019). A small number of studies suggest that focal lesions near somatotopic hand motor areas diminish accuracy in the HLJ task (Argiris et al., 2020; Tomasino et al., 2011) but not in action naming and semantic relatedness judgements (Argiris et al., 2020). Unfortunately, RTs were not reported in the latter study which are likely to be more sensitive to smaller processing deficits.

Moreover, even though motor execution and action-specific conceptual deficits tend to correlate, there are individual cases in which action execution and action knowledge dissociate (Negri et al., 2007; Papeo et al., 2010; Rapcsak et al., 1995; Vannuscorps et al., 2016): e.g., impaired action execution but spared action knowledge. This result is comptabile with two opposing scenarios: 1) Separate systems underlie action production and comprehension (e.g., Negri et al., 2007). On this view, associations between the two arise when both the production infrastructure and action-specific aspects of the comprehension system are damaged. This would be expected to be common if they are proximal to each other, as suggested in the “anterior shift hypothesis” (see Mahon, 2015; Thompson-Schill, 2003). Dissociations arise in cases where only one of the components is impaired. 2) The action production system is functionally relevant for conceptual processing (i.e., production and comprehension systems are not separate). However, multiple systems are available to solve tasks tapping conceptual processing of actions. For instance, accessing information about visual motion is likely to be important for task performance (Watson et al., 2013). There are individual differences regarding the dominance of particular sources of information, as well as regarding the ability to use alternative sources of information when one is unavailable due to brain damage. Thus, some patients with apraxia may achieve good task performance due to the compensatory use of alternative processes (see Barsalou, 2016).

**Figure 3:**
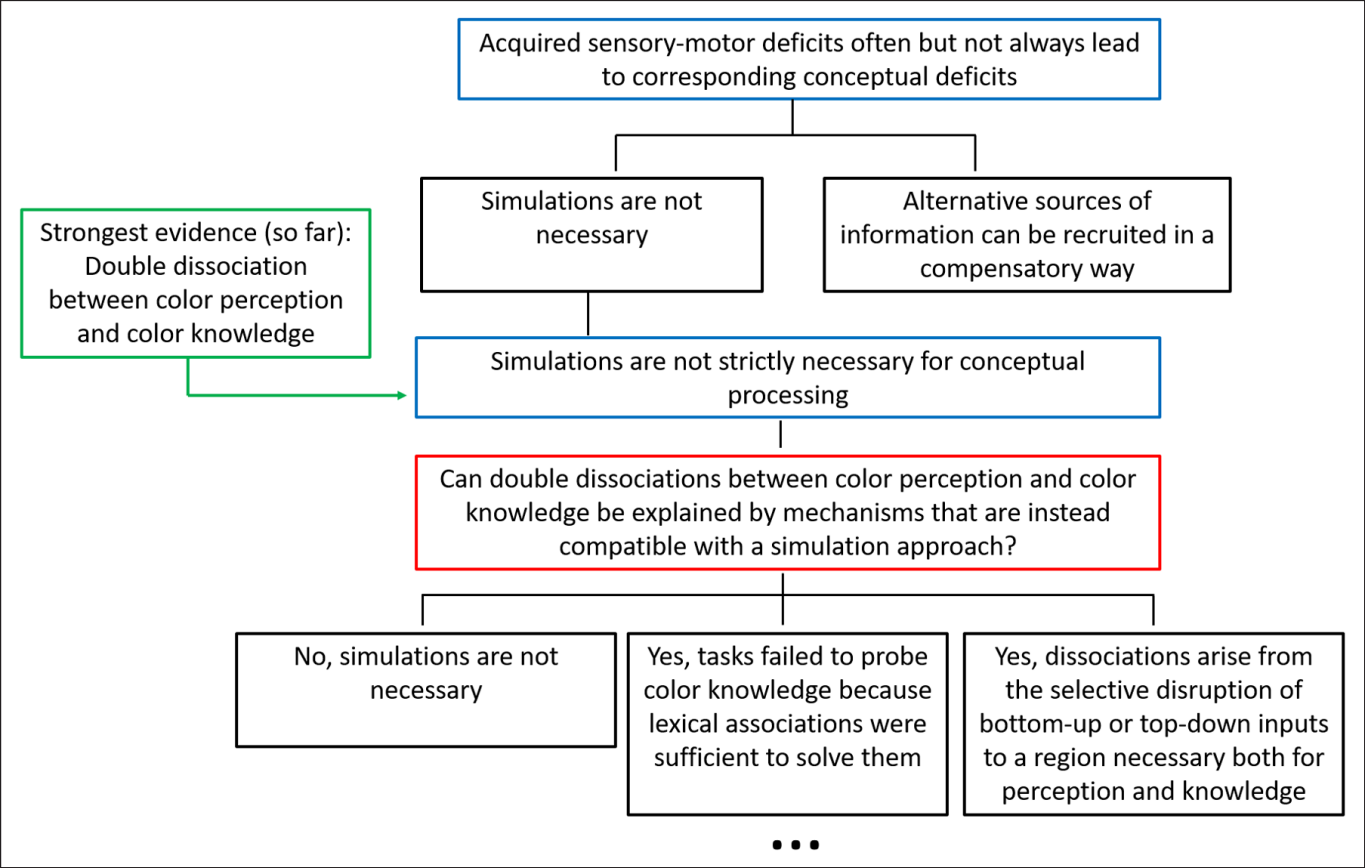
Logical tree illustrating alternative hypotheses compatible with the pattern that acquired sensory-motor deficits often but not always lead to corresponding conceptual deficits.

One possible way to adjudicate between these two proposal could be, once again, to look into conceptual features that are unimodal and hardly re-mappable to alternative types of experience, such as color. For instance, a patient with damage in the visual cortex that impairs color perception (achromatopsia) but leaves color knowledge unchanged would be hard to explain on the bases of the use of alternative simulations that are spared by the brain damage. Whereas associations between color perception and color knowledge are common (De Renzi & Spinnler, 1967; Farah, 1989), examples of dissociations have been reported, which appear to suggest that simulation is not necessary for color knowledge retrieval. In particular, patients with intact color perception but impaired color knowledge have been observed (Miceli et al., 2001; Stasenko et al., 2014), as well as patients with intact color knowledge but impaired color perception (Shuren et al., 1996). However, once again, in addition to the inuitive interpretation (here, that color perception and knowledge rely on separate cortical substrates), two additional alternatives to this conclusion have been proposed (see ***Figure 3***): 1) Patients may *appear* to have normal access to color knowledge even though they do not, if tasks are used in which lexical associations suffice to solve them (for example, the words *grass* and *green* are strongly associated). Patients may fail when this strategy is ruled out (Beauvois & Saillant, 1985). 2) Dissociations are compatible with a scenario where a color perception region is necessary for color perception and access to color knowledge under the following assumption: Disruptions to bottom-up inputs to that region lead to color perception deficits and disruptions to top-down inputs to the same region lead to color knowledge deficits (De Vreese, 1991; Shuren, Brott, Schefft, & Houston, 1996). Simultaneous disruptions to bottom-up and top-down inputs, or damage to that region itself, lead to color perception and color knowledge deficits. This sort of model can not only account for the general dissociations and associations just described, but also for category-specific color knowledge deficits. This would be due to disruptions to connections between regions important for a certain category and the color perception region (Stasenko et al., 2014). Alternative 1 can be straightforwardly tested by contrasting patients’ performance on tasks that can vs. cannot be solved by simply relying on lexical associations. Alternative 2 can be addressed with meta-analyses of data from patients mapping out which regions and cerebral connections are associated with which behavioral outcomes.

**Figure 4:**
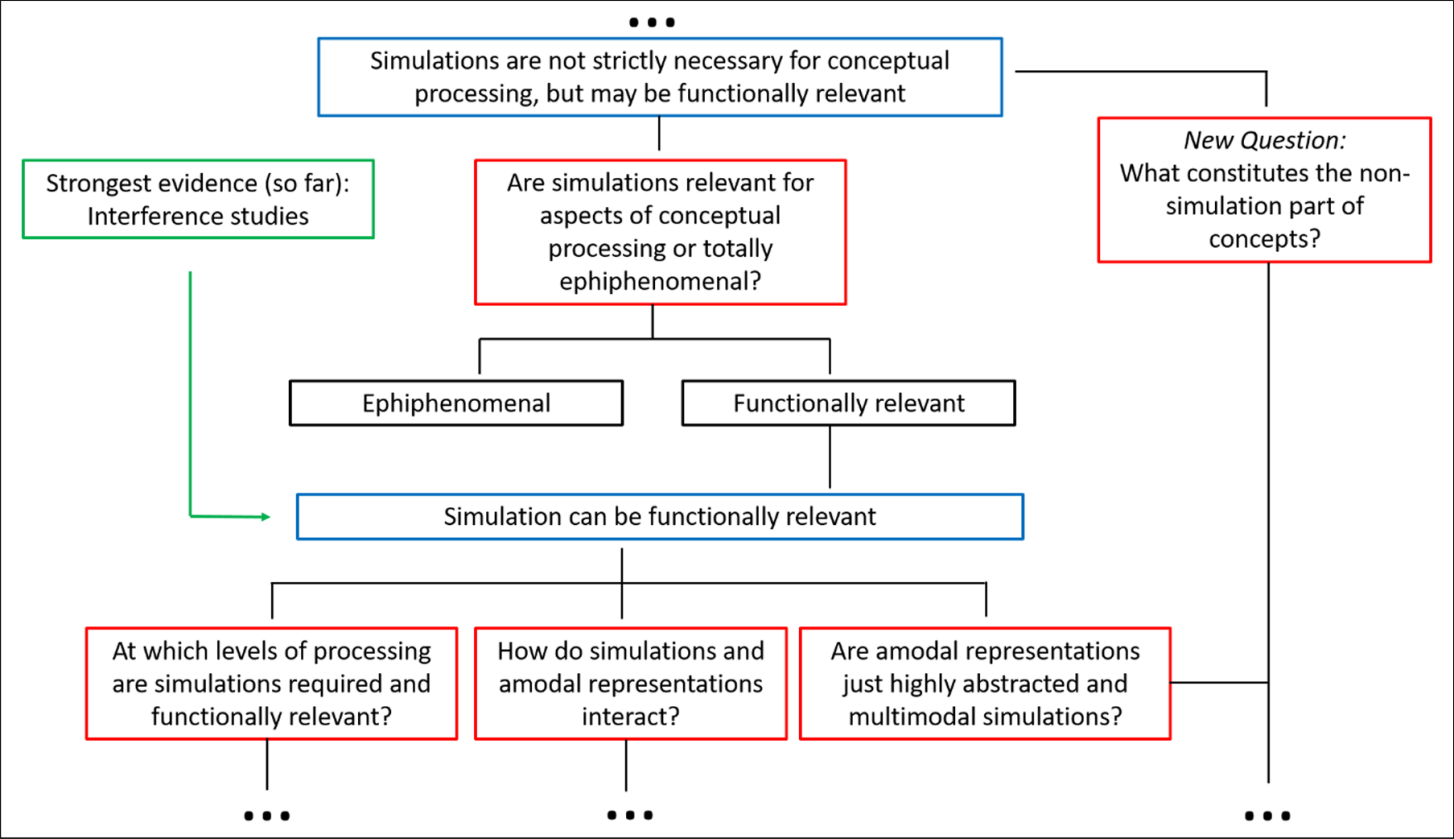
Third part of the logical tree specifying more detailed questions on the role of simulations.

The upshot of all this is that color is a very promising domain to solve the functional role of simulations for conceptual processing, but the available empirical data do not answer it yet. This is not to say that no progress has been made. To the contrary, patient studies have strongly constrained the possibility space such that targeted investigations can hope to come ever closer to solving the puzzle of conceptual knowledge and sensory-motor simulation. For instance, recent studies presented evidence from a rare patient with damage to the left ventral occipital-temporal cortex, who has spared color perception, can state which color an object typically has, and can tell whether an object is typically colored or not, but has a strong impairment for color naming, matching color names to color patches or typically colored objects, and matching a color patch to the memory color of a greyscale object (Siuda-Krzywicka et al., 2019a; b). What all of the impaired tasks have in common is that it is necessary to abstract color information from objects. Thus, for fully-fledged color knowledge perceptual representations might not be enough and language (i.e., intact connectivity pathways with anterior-temporal regions) may be required for an abstract representation of color that has been suggested to otherwise be bound to holistic object representations in the visual system (Siuda-Krzywicka et al., 2019b). This very interesting result suggests that certain levels of knowledge cannot be based solely onto sensorimotor simulations, and potentially opens a new branch on our logical tree, asking what exactly constitutes the non-simulation part of concepts (***Figure 4***).

In sum, based on the available evidence, acquired sensorimotor disorders due to brain damage cannot yet corroborate strong inference regarding the strict necessity of sensory-motor simulations for conceptual processing or regarding their functional role. However, they hold promise for providing important evidence in the future, especially when unimodal conceptual domains are used as a test-bed, and are likely to open up new interesting questions.

### 2.3. INTERFERENCE PARADIGMS WITH HEALTHY PARTICIPANTS

The previous sections showed that studying the effects of congenital and acquired sensory-motor impairments can bring valuable and unique insights into the mind and brain. However, this approach might not be enough to decide about the functional relevance of simulations during conceptual processing. When the possibility of compensation, plasticity or “remapping” is minimized (as in the case of color for congenitally blind or achromatopsic patients), congenital and acquired sensory deprivation can adjudicate whether or not simulations are strictly necessary for conceptual processing, but for now they left open the question of their functional relevance when in place. When simulations are possible and used, are they functionally relevant for conceptual processing?

Luckily, researchers have a powerful tool at their disposal that can complement patient studies and overcome many of their limitations. Interference paradigms with healthy participants have the potential to bring about substantial progress. They are much easier to carry out and share most of the appealing aspects of patient studies. This makes them ideal from the perspective of strong inference because series of studies can feasibly be conducted that progressively exclude alternatives until one reaches a clear answer. Yet, in our view their unsystematic use has led to rather slow progress to-date. The advantages of interference paradigms are very similar to those of patient studies. As Mahon and Caramazza (2008, p. 64) recognized, compared to paradigms that test the mere activation of sensory-motor processes, “a stronger test of the embodied cognition hypothesis would consist in studying perceptual and conceptual processing subsequent to suppression or impairment of the motor system.” This can be achieved with TMS or behavioral interference techniques. In comparison to congruency or neuroimaging paradigms, which can address whether sensory-motor systems are *activated* in a given task, interference paradigms tap into the crucial question of *functional relevance*.

Several studies have used simple interference paradigms to test the causal role of motoric processes for online conceptual processing. For instance, squeezing a ball was found to slow down the naming of tools whose handles are oriented towards the squeezing hand and made it less accurate when pictures where presented very briefly (Witt et al., 2010). Similarly, performing nonsense actions reduced naming accuracy of manipulable objects (but had no effect on RTs) and it reduced accuracy in concreteness judgements to nouns referring to manipulable objects (Yee et al., 2013). A robust effect of the same motor interference technique on RTs was recently reported in a high-powered experiment (N = 198) using an animacy judgement task, and the amount of interference was directly related to the amount of motor experience associated with a given concept (Davis et al., 2020, Experiment 2). In contrast, Strozyk et al. (2019) found no effects of hand or foot tapping on the processing of corresponding verbs in a LDT. This pattern of results is compatible with the view that interference effects were observed in semantic tasks that require access to specific referential information. However, this conclusion is tentative at this point and it would be useful for researchers to systematically evaluate the role of experimental factors (timing, type of interference, task) in order to produce maximally interpretable results.

A potential concern with such motor interference paradigms is that squeezing a ball might not only engage the motor system, but also high-level conceptual representations. For instance, squeezing a ball might activate action concepts, such as SQUEEZE or HAND, influencing conceptual processing in a category-specific way. Thus, one might argue that these paradigms run into the same interpretational ambiguity we described for classic congruency effects (e.g., Glenberg & Kaschak, 2002; Zwaan et al., 2002). However, it is not clear why the activation of related concepts would hinder, as opposed to facilitate, processing. In word comprehension, negative semantic priming has only been reported in very particular settings, such as in selective attention tasks in which target stimuli are embedded in distractor stimuli (D’Angelo et al., 2016). These paradigms are very different from the interference paradigms we discussed above and it seems unlikely that they are due to semantic interference at an amodal level.

Nevertheless, to avoid this sort of ambiguity it would be ideal to use interference techniques that are not associated with (task-relevant) concepts in the first place. This can be achieved in the visual domain, where nonsense masks can be used to interfere with visual simulation. The visual properties of meaningless masks (visual noise) can be selected so as to interfere with particular types of processes in the visual system. Several studies have obtained evidence that interfering with basic visual processes during semantic processing specifically diminishes access to information about visual features of word referents. Dynamic low-level visual noise interfered with the processing of concrete relative to abstract words in a concreteness judgement task, but not in a lexical decision task and a word class judgement task (Ostarek & Huettig, 2017a). In the same vein, low-level visual noise reduced the effectiveness of word cues in a word-picture verification task and it worsened performance in a property verification task probing visual (table – is it round?) but not categorical (table – is it furniture?) knowledge (Edmiston & Lupyan, 2017). Static masks with visual features that are specifically potent in interfering with the visual perception of pictures were shown to also interfere with the categorization of a sound as animate or inanimate (Rey et al., 2015). Strikingly, after participants learned to associate a tone with such a visual mask, simply hearing the tone interfered with semantic categorization of spoken words (animal vs. artifact) as a function of the perceptual strength of the word (Rey et al., 2017). Finally, a recent high-powered study further demonstrated the experience-dependent recruitment of sensory-motor processes during semantic processing (Davis et al., 2020). Participants (N = 205) performed animacy judgements on words that varied in the extent to which their referents were associated with visual experience while performing a concurrent visual task on nonsense shapes. The key result was that the amount of interference that the visual task excerted was proportional to how much visual experience was associated with a given concept (Davis et al., 2020, Experiment 1). Thus, in simple paradigms involving single word processing, there is extraordinary convergence of evidence strongly suggesting that visual processes contribute functionally to conceptual/semantic processing specifically when the retrieval of visual information is task-relevant. It is important to note that all the studies above used visual noise composed of meaningless shapes. Neuroimaging data suggest that such masks selectively activate the visual cortex (Yuval-Greenberg & Heeger, 2013). Thus, the issue with typical congruency pardigms, where it is unclear at which level effects arise, does not apply.

The highly useful visual noise technique has barely been extended beyond single-word processing paradigms. One study (Ostarek, Joosen, et al., 2019) evaluated the functional role of visual simulation in sentence processing, relying on the sentence-picture verification paradigm (Zwaan et al., 2002). In this paradigm, participants process a sentence implying an object to be in a particular shape and then have decide whether a picture appearing immediately after the sentence depicts an object that was mentioned or not. Typically, RTs are shorter to images that match the shape implied in the sentence. This is often interpreted as evidence for visual simulation (Zwaan et al., 2002; Zwaan & Pecher, 2012). The main result was that various types of visual noise, ranging from low-level to high-level visual, did not robustly diminish the typically observed advantage for targets with shapes congruent with the shape implied in the sentence. This suggests that the shape match effect does not depend on visual simulation (Ostarek, Joosen, et al., 2019). Clearly, more interference studies are required to elucidate whether combinatioral language processing relies on visual simulation.

Behavioral interference paradigms have also been applied to the domain of working memory. A behavioral interference study by Shebani and Pulvermüller (2013) suggests that complex hand or foot movements can lead to effector-specific interference with working memory for corresponding action verbs. Similarly, Downing-Doucet and Guérard (2014) reported that opening and closing the hand during encoding interfered with memory for graspable objects (see also Lagacé & Guérard, 2015). Moreover, a series of experiments consistently showed that the retrieval of manual actions or manipulable objects held in short-term memory was impaired when participants had to hold their hands behind the back during encoding, whereas memory of non-manual actions or non-manipulable objects was not affected by posture (Dutriaux et al., 2019; Dutriaux & Gyselinck, 2016). By contrast, a number of largely similar studies did not obtain any evidence that motor interference affects working memory for action-related objects (Canits et al., 2018; Pecher, 2013; Quak et al., 2014; Zeelenberg & Pecher, 2016). At this point, it is not clear whether or when motor interference causes action-specific working memory impairments (Montero-Melis, van Paridon, Ostarek, & Bylund, 2019; Zeelenberg & Pecher, 2016).

Researchers investigating effects of action language on movements are faced with a similarly heterogeneous picture that makes it hard to draw firm conclusions yet. A review of 108 experiments proposes that it is possible to predict whether action language facilitates, interferes with, or has no effect on movements based on temporal and task-related factors (García & Ibáñez, 2016). Systematic analyses like this are crucial to reach a situation where more and more specific questions can be addressed in new empirical studies.

Transcranial magnetic stimulation (TMS) is another prominent interference technique. TMS pulses are particularly easy to administer to effector-specific parts of the motor cortex, due to its somatotopic organization. However, the TMS literature tapping into motor simulation is rather small and does not paint a coherent picture. This is particularly striking in the case of action language comprehension, where a variety of stimulation protocols have been used and a variety of different results have been obtained. Shorter RTs to action verbs were obtained in a lexical decision task when two-pulse TMS was delivered online to the hand M1 region (Pulvermüller et al., 2005) and when theta-burst TMS was delivered offline to the premotor hand area (Willems et al., 2011). Another set of studies, by contrast, observed longer RTs after TMS of the motor cortex. In a concreteness judgement task, RTs to action verbs were increased after online four-pulse TMS on hand M1 (Vukovic et al., 2017) and after offline repetitive TMS on M1 (Repetto et al., 2013). Vukovic et al. observed no effects of TMS when a lexical decision task was used. This tentatively suggests that TMS on the motor cortex is more likely to hinder processing when action-related information is conducive to task performance, such as in the concreteness task or in motor imagery tasks (Tomasino et al., 2008). However, another study reported no effects of TMS on M1 when participants were asked to decide whether verbs referred to actions or not (Papeo et al., 2009), which arguably directly probes action knowledge. A further study found that repetitive TMS on M1 selectively slowed down responses to action verbs and action-related nouns in a morphological transformation task (Gerfo et al., 2008). Overall, it is interesting that several papers have reported effects of TMS of the motor cortex. But it is hard to see which factors determine whether an effect is expected and which sign the effect will have. An important challenge will be to reach a situation where specific predictions can be made (Ostarek & Huettig, 2019). So far, the literature is very much unlike the ideal scenario that Platt envisioned for strong inference (Platt, 1964), where one factor after the other (e.g., number, timing, intensity of TMS pulses, task, etc.) is systematically probed until the picture becomes clear.

An additional issue is that TMS of a given region can have distant effects on functionally connected regions (e.g., Ruff et al., 2009). The logic of many TMS studies is that stimulating a given area should only impact processing if the area carries out processes that are functionally relevant. However, it is possible that indirect stimulation of a functionally connected region accounts for the observed behavioral effects. For instance, there is evidence that the posterior middle temoporal gyrus is functionally connected to the motor cortex and that stimulating the former can alter processing in the latter (Papeo et al., 2015). Conversely, it is conceivable that stimulation of the motor cortex can influence processing in temporal regions. Thus, It will be crucial to understand how different nodes of the conceptual system interact. This could be addressed with dual-site TMS that stimulates different nodes at different points in time (Hartwigsen et al., 2010).

In sum, behavioral interference methods hold great promise to elucidate the functional role of sensory-motor simulation for cognitive functions such as language comprehension, but their utilization so far does not live up to the potential of the method. They lend themselves rather ideally to systematically tackling open questions in the debate about embodiment one by one in a highly targeted way, in the spirit of strong inference (Platt, 1964). This should be done in the context of pre-registration, to decrease researcher degrees of freedom (Simmons et al., 2011), and open science, to alleviate the file drawer problem and make research more reproducible (Nosek et al., 2015).

## 3. CONCLUSION

Research on embodiment has not yet resolved essential issues necessary for the field to take the next step. Here, we proposed that this is partly due to the prominent reliance on weak paradigms that do not allow for strong theoretical inferences. Causal paradigms can deliver strong inference regarding an essential prediction of embodied accounts according to which experience-sensitive sensory-motor simulations functionally contribute to cognitive processing.

In discussing the explanatory potential of causal paradigms, we focused on congenital sensory-motor disorders, acquired sensory-motor deficits, and interference paradigms with healthy participants. Participants with congenital sensory-motor disorders can provide unique insights into the scope and limits of neural plasticity. Comparisons with healthy participants in terms of task performance are challenging because it is typically unclear whether equivalent performance is due to lacking functional relevance of sensory-motor simulation or due to experience-based adaptation/compensation. It is likely to be fruitful to contrastively investigate domains where alternative sensory experience is available (blind people can experience tools via touch) with those that can uniquely be experienced via the unavailable sense (color can only be seen). Experience-based accounts clearly predict processing differences in the latter case. Such differences can manifest themselves in behavioral measures and/or in altered neural processing which can be tested with modern neuroimaging methods. Studying acquired sensory-motor deficits can potentially be revealing about to role of simulations in conceptual processing, although at the moment they do not provide strong inference yet. Interference paradigms with healthy cohorts have the potential to be decisive in the debate about embodiment. They tend to be cheap, can be administered to large samples of healthy participants, afford great experimental control, and can be implemented in a large variety of tasks. When researchers wish to make claims about embodiment, a general switch from using congruency paradigms to using interference paradigms as a default could be a crucial move.

We examined three main alternatives regarding the role of simulations in conceptual processing: they are strictly necessary and functionally relevant, they are not srictly necessary but functionally relevant, they are neither strictly necessary nor functionally relevant. Our analyses suggests that we can reasonably exclude the possibility that simulations ar strictly necessary for conceptual processing on the basis of spared color knowledge in some congenitally blind individuals. Future research can move on to more fine-grained questions regarding, for instance, the extent and origins of color knowledge in congenitally blind people.

Second, we can exclude the possibility that simulations are neither strictly necessary nor functionally relevant. This is mainly based on results from interference paradigms, which have made a convincing case for the view that experience-based simulations contribute functionally to conceptual processing (Davis et al., 2020; Edmiston & Lupyan, 2017; Ostarek & Huettig, 2017b). This line of research has made a strong case for basic visual processes being functionally relevant as a function of the sensory experience associated with a concept and as a function of the relevance of perceptual information in a given task. Important open questions are whether this pattern also holds in other modalitites (e.g., motor) and whether simulation is functionally relevant in typical communication situations (see Ostarek & Huettig, 2019 for discussion).

The main goal of this paper was to illustrate how a focus on strong inference and causal paradigms can generally be beneficial. To do so in an amount of pages that we can expect readers to tolerate, we painted in rather broad strokes. For instance, ultimately the real challenge is not to determine whether simulations contribute functionally to high-level cognition in general, but to figure out whether simulations underlie specific functions or domains. In a similar vein, the ultimate goal is not to determine whether sensory-motor simulations (of any kind) are important, but to understand which exact processes are activated in a given task. Thus, the idea of the current paper was not to do all the heavy lifting already, but to propose an approach that we deem promising to get a good grip on the question of embodiment. Finer-grained questions should follow naturally by going deeper in the logical trees we have sketched, by adding new branches etc. We also welcome a discussion of whether the approach based on strong inference we presented here can be improved, or whether an altogether-different approach is superior.
